# Parasitism, seasonality, and diversity of trombiculid mites (Trombidiformes: Parasitengona, Trombiculidae) infesting bats (Chiroptera) in Poland

**DOI:** 10.1007/s10493-021-00683-7

**Published:** 2021-12-07

**Authors:** Paula Zajkowska, Joanna Mąkol

**Affiliations:** Department of Invertebrate Systematics and Ecology, Wrocław University of Environmental and Life Sciences, Kożuchowska 5b, 51-631 Wrocław, Poland

**Keywords:** *Leptotrombidium* spp., Morphology, COI, Phenology, Host range, Attachment sites, Chiggers

## Abstract

The study aims to ascertain the diversity of trombiculid species associated with Chiroptera in Poland, and for the first time in the case of research on Central European Trombiculidae, we use both DNA and morphology in an integrative taxonomic approach to determine species identities of trombiculids. The research was carried out from 2015 to 2019. In total, 2725 larvae were collected from 300 specimens of bats belonging to 11 species. Deutonymphs were obtained through laboratory rearing of larvae; few larvae and deutonymphs were collected also from bats' daily roosts. The presence of trombiculid larvae on hosts was observed between July and April of the following year, with the highest numbers recorded in autumn, during bat swarming. Male bats were infested more often than females (16.4 vs. 6.6%). The highest infestation rate was recorded for *Barbastella barbastellus*, *Myotis nattereri* and *Plecotus auritus*, and the highest prevalence of chiggers (> 30%) for *Myotis bechsteinii* and *P*. *auritus*. The larvae found on bats occupied the areas with free access to the host’s skin: auricles, tragus, and snout. Morphological identification of specimens to the species level was hindered by the mosaic distribution of diagnostic traits. Morphological analyses indicated the presence of *Leptotrombidium russicum* and *Leptotrombidium* spp. in the examined material, whereas molecular analyses additionally suggested three other potential species assigned to the same genus based on the assessed scope of intrageneric variation (ASAP method). We argue that the identification of the parasitic larvae (chiggers) using morphological characters does not address the question of actual species boundaries, which, in turn, affects the inferences about host specificity and host range.

## Introduction

Trombiculidae sensu Kudryashova (1998), with more than 3000 nominal species worldwide (Liu et al. 2013; Nielsen et al. 2021), is the most species-rich family within terrestrial Parasitengona mites. Most species, recognized based solely on morphological criteria, are known exclusively from larvae, which parasitize various vertebrate and a few invertebrate hosts (Nadchatram 2006; Wohltmann et al. 2006; Shatrov and Kudryashova 2008; Stekolnikov and Kar 2015; Stekolnikov et al. 2016; Caputo et al. 2018; Felska et al. 2018; Kaya and Yilmaz 2019). Despite the large number of papers related to Trombiculidae (Actinotrichida: Parasitengona) published during the last 80 years, knowledge about ecological demands and biology of species remained scarce. This is in particular true in case of chiggers associated with bats (Chiroptera).

More than 400 nominal species of chiggers have been reported as parasites of Chiroptera worldwide (Zajkowska et al. 2018; Bassini-Silva et al. 2021; Kalúz et al. 2021; Ševčík et al. 2021), but only two of them—*Leptotrombidium russicum* (Oudemans) and *Oudemansidium musca* (Oudemans)—have been recorded from bats in Poland (Moniuszko and Mąkol 2014). A roughly similar representation of bat-associated chiggers has been noted in other Central European countries, e.g., the Czech Republic [*L. russicum* and *O. musca* but also *Oudemansidium komareki* (Daniel & Dusbábek), *Neotrombicula autumnalis* (Shaw) and *Neotrombicula japonica* (Tanaka et al.)], Slovakia (*L. russicum*, *O. musca*, *O. komareki*), and Hungary (*L. russicum*) (Zajkowska et al. 2018). Both *N. autumnalis* and *N. japonica* have also been recorded from Poland, but the findings referred to associations of these species with rodents, soricomorphs, and in the case of *N. japonica*, also carnivores (Moniuszko and Mąkol 2014).

So far, 1402 bat species belonging to 21 families have been described in the world (Wilson and Mittermeier 2019). In Poland, the presence of 27 species (1.9% of the world's fauna of bats), aggregated in the families Vespertilionidae (24 species), Rhinolophidae (two species) and Miniopteridae (one species), has been confirmed (Okarma et al., https://www.iop.krakow.pl/Ssaki/gatunki; Piksa and Gubała 2020). Of those, as many as 14 species have been hitherto recorded in Poland as hosts of trombiculid larvae of the genera *Leptotrombidium* (*L. russicum*) (12 bat species) and *Oudemansidium* (*O. musca*) (four bat species) (Willmann 1952; Harmata 1967; Haitlinger and Ruprecht 1977, 1985, 1992; Haitlinger 1979; Haitlinger and Łupicki 2008; Moniuszko and Mąkol 2014).

According to some authors (e.g., Shatrov and Kudryashova 2006), the host specificity of trombiculid mites is extremely low, and habitat preferences rather than phylogenetic affiliation of the host plays a crucial role in host selection. The number of host species (host range) exploited by these parasites is deemed to become defined at higher taxonomic levels; however, the frequency of infestation (prevalence) of a given host species remains unknown. The actual host specificity may be negatively influenced by incorrect identification of the trombiculid larvae, especially when based exclusively on morphological evidence. Nevertheless, of all hitherto recognized associations between chiggers and their hosts, the genera and species which exploit bats are considered the most host-specific (Shatrov and Kudryashova 2006; Stekolnikov and Quetglas 2019).

The present study aims to ascertain the diversity of trombiculid species associated with Chiroptera in Poland, using both morphological and molecular criteria, which should translate to improved understanding of host range and host specificity of bat-associated chiggers.

## Materials and methods

### Sampling

The field work was carried out from 2015 to 2019. All bats were caught under permits (DZP-WG.6401.09.05.2015.km.7, DZP-WG.6401.09.12.2016/2017.dł.2) issued by the General Directorate for Environmental Protection and within the frame of cooperation with Polish chiropterologists. Trapping of bats was conducted using mist nets and/or a harp trap (1.5 × 2 m). Each bat was temporarily transferred to a cotton bag, to avoid cross-contamination with trombiculid larvae. In ascertaining the taxonomic affiliation and sex of bats the identification key to the bats of Europe was used (Dietz and von Helversen 2004). In winter, due to the limited access to hibernacula and the restrictions in capture of bats, the larvae were obtained only from available hosts, in compliance with bat protection rules.

Most trombiculid larvae removed from the hosts using smooth forceps, bent at 45°, were transferred directly to EtOH. The larvae at the highest level of engorgement were placed in rearing vials (25 × 35 mm glass containers, with semi-transparent lid). To obtain active postlarval forms for morphological analyses, laboratory rearing was carried out, within the parallel and independent experiment testing the development success to subsequent instars at laboratory conditions. Additionally, we searched the daily roosts of bats for engorged larvae that had dropped off their hosts as well as for representatives of active postlarval stages. For that purpose, 100-mL containers filled with glycerol or EtOH were placed under/between loose chunks of bark and in frost cracks.

### Parasitological indices and attachment sites

To determine the general parameters related to the level of infestation with trombiculid larvae, the prevalence, mean intensity and mean abundance (Bush et al. 1997; Whitaker et al. 2009) were estimated. Descriptive statistics, calculated collectively for ectoparasitic chiggers collected during the survey, were computed in R software (R Core Team), with application of R Studio (v.1.2.5033). In ascertaining the statistical significance of differences in infestation of bats’ females and males the Mann–Whitney U test was applied. Differential use of attachment sites on the of host's body was analyzed in a descriptive manner.

### Morphological and molecular identification of Trombiculidae

One hundred mites randomly selected from all samples (larvae collected from different host species, deutonymphs obtained by experimental rearing from larvae taken from hosts, deutonymphs and larvae collected from the daily roosts of bats) were subject to detailed morphological analyses. The material was mounted on microscopic slides in Faure’s fluid (Walter and Krantz 2009). Measurements were taken using a Nikon Eclipse E600 compound microscope, equipped with differential interference contrast (DIC) and DS-Fi1 camera system, using the NIS-Elements BR software (https://www.microscope.healthcare.nikon.com/en_EU/products/software/nis-elements/nis-elements-basic-research). To identify larvae to genus and species we used various identification keys (Kudryashova 1998; Fernandes and Kulkarni 2003; Stekolnikov 2013), and in the case of deutonymphs, the original descriptions as well as redescriptions of species (Kepka 1959; Crossley 1960; Mąkol et al. 2010) served as a source.

For molecular analysis we used a non-destructive method of DNA extraction (Cruickshank 2002; Dabert et al. 2008; Porco et al. 2010) aimed at retaining the exoskeletons for morphological examination. The DNA extraction and polymerase chain reaction protocols (Bernard et al. 2019) were applied with the following modifications: only the bcdF01 primer and 12.5 µl of KAPA2G Robust HotStart ReadyMix were used; the annealing temperature for PCR was 49 °C. PCR assay was applied to amplify the fragment of the mitochondrial cytochrome c oxidase subunit I gene (COI). The amplification product was sequenced in both directions (Genomed, Poland). The obtained nucleotide sequences were analysed using the MEGA X program (Stecher et al. 2020) and Geneious v.9 (Kearse et al. 2012). The sequences are deposited in the GenBank (for accession numbers see Table 1, rows 5–8). The sequences for additional trombiculid species and outgroup taxa (Table 1, rows 1–4, 9–20) were retrieved from GenBank.

**Table 1:** Taxa and corresponding COI sequences applied in the phylogenetic analyses and in the ASAP method

	Species [distribution^a^]	Accession number [GenBank]	Country (collection site)	References
1	*Hirsutiella zachvatkini* (Schluger) [PAL]	KR071845	Poland	Moniuszko et al. (2015)
2	*Miyatrombicula muris* (Oudemans) [PAL]	MH622154	Poland	Moniuszko et al. (2018)
3	*Neotrombicula inopinata* (Oudemans) [PAL]	MH607466	Poland	Moniuszko et al. (2018)
		KR337639	Spain	Santibáñez-Sáenz (2015)
4	*Neotrombicula vulgaris* (Schluger) [PAL]	KY888693	Poland	Moniuszko et al. (2017)
5	*Leptotrombidium russicum* (Oudemans) [PAL]	OL619429, OL619430, OL619431	Poland	This study
6	*Leptotrombidium* sp. 1	OL619433, OL619434, OL619435	Poland	This study
7	*Leptotrombidium* sp. 2	OL619436	Poland	This study
8	*Leptotrombidium* sp. 3	OL619432	Poland	This study
9	*Leptotrombidium akamushi* (Brumpt) [ORI; PAL; AUS]	NC007601	Japan	GenBank^b^
10	*Leptotrombidium fletcheri* (Womersley and Heaslip) [AUS; ORI]	AB300489	No data	GenBank^b^
11	*Leptotrombidium imphalum* Vercammen-Grandjean and Langston (syn. *L*. *chiangraiensis* Tanskul and Linthicum) [ORI; PAL]	HQ324935, HQ324944, HQ324949, HQ324965, HQ324968, HQ324969, HQ324971, HQ324972	Thailand	GenBank^b^
12	*Leptotrombidium deliense* (Walch) [ORI; PAL; AUS]	HQ324977	No data	GenBank^b^
		KY930745, KY930749, KY930750, KY930751	Laos	Kumlert et al. (2018)
		MH446370	Thailand	GenBank^b^
13	*Leptotrombidium scutellare* (Nagayo, Miyagawa, Mitamura and Tenjin) [PAL]	AB300498	No data	GenBank^b^
14	*Leptotrombidium palpale* (Nagayo, Miyagawa, Mitamura and Tamiya) [PAL]	AB300499	No data	GenBank^b^
15	*Leptotrombidium pallidum* (Nagayo, Miyagawa, Mitamura and Tamiya) [PAL]	AB180098	Japan	Shao et al. (2005)
16	*Leptotrombidium intermedium* (Nagayo, Mitamura and Tamiya) [PAC; PAL]	AB300492	No data	GenBank^b^
17	*Leptotrombidium* sp.	AB300494	No data	GenBank^b^
18	*Leptotrombidium fuji* (Kuwata, Berge and Philip) [PAL]	AB300496	No data	GenBank^b^
19	*Bdellidae* sp. 1	KM100983	No data	Dabert et al. (2016)
20	*Bdellidae* sp. 2	KM100984	No data	Dabert et al. (2016)

^a^distribution by zoogeographic region after Nielsen et al. (2021): *AUS* Australian Region, *PAC* Pacific Region, *PAL* Palearctic Region, *ORI* Oriental Region

^b^sequences retrieved from GenBank; authors and year of submission available in GenBank

The mites used for morphological analyses, including exoskeletons retained after DNA extraction, are deposited in the acarological collection of the Department of Invertebrate Systematics and Ecology, Wrocław University of Environmental and Life Sciences.

### Species delimitation and phylogenetic analyses

For species delimitation of our specimens and those represented by sequence data in GenBank, we applied the default options of Assemble Species by Automatic Partitioning (ASAP) method (Puillandre et al. 2021), available at https://bioinfo.mnhn.fr/abi/public/asap. Genetic distances among sequences were estimated with Kimura-2 parameter (K2P) substitution model (Kimura 1980). The ASAP inference was used for trombiculid sequences derived from specimens collected from bats, bat roosts and from *Leptotrombidium* spp. sequences filed in the GenBank database (Table 1, rows 5–18). The partitions with the best asap-score (lowest value) were selected.

The Bayesian phylogenetic inference (BI) was performed with MrBayes (Ronquist and Huelsenbeck 2003; Ronquist et al. 2012) using Markov chain Monte Carlo (MCMC) algorithm and with the application of the following commands: ngen = 1000000, samplefreq = 1000, sump/sumt burnin = 250. The phylogenetic tree visualization was made in FigTree v.1.4.4 (Rambaut 2018).

## Results

**Fig. 1:**
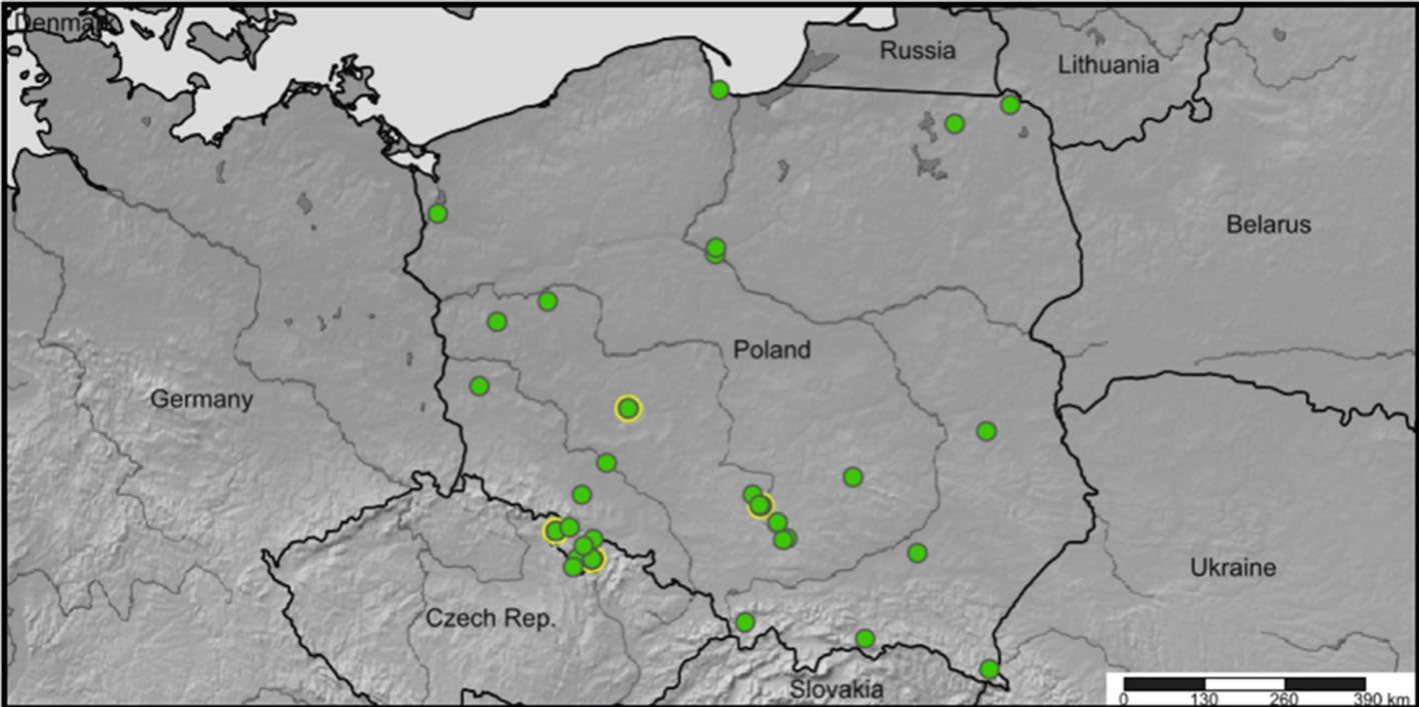
Collecting sites of bat-infesting chiggers in Poland (36 localities recorded during present survey, in 2015–2019). Green dots: Trombiculidae; green dots with a yellow margin: *L. russicum*. (Color figure online)

The localities in which the ectoparasitic larvae were found on hosts covered the area spreading out between 49°12′0.194″N–54°23′43.685″N latitude and 14°36′53.64″E–22°53′20.399″E longitude, within the administrative borders of Poland (Fig. 1).

### Prevalence, intensity, and abundance

Out of 2813 bats from 19 species, chiggers were observed on 300 individuals (10.7%) from 11 species (Table 2). Larvae were not recorded on *Eptesicus nilssonii*, *Myotis dasycneme*, *Myotis emarginatus*, *Myotis mystacinus*, *Nyctalus noctula*, *Pipistrellus nathusii*, *Pipistrellus pipistrellus*, and *Vespertilio murinus*, for which a total of 474 specimens were caught. Male bats constituted 81.3% of all infested host individuals and 16.4% of males representing species confirmed as hosts were parasitized by chiggers; the respective values for females were lower (18.7 and 6.6%). Differences in infestation of females and males were significant (Mann–Whitney U test: W = 4887.5, P = 0.008). The only exception to a male bias in infestation was *Rhinolophus hipposideros*, in which, contrary to other species, the males were not infested; note, however, that the overall number of examined males was (much) lower than that of females for this species (Table 2). Altogether, 2725 trombiculid larvae were collected from these hosts. The highest infestation rate (prevalence) was recorded for *Barbastella barbastellus*, *Myotis nattereri* and *Plecotus auritus*, whereas the highest prevalence of chiggers (> 30%) was recorded for *Myotis bechsteinii*, *P*. *auritus* and *P*. *austriacus* (Table 2); however, in the case of *P*. *austriacus* the very high prevalence was due to a single individual of this species being infested.

**Table 2:** Recorded host species and values of parasitological indices referring to trombiculid mites collected during the survey in Poland (2015–2019)

	Host species	No. bats examined	No. infested specimens/prevalence	Total no. larvae collected from hosts	Mean (± SD) intensity	Mean abundance	Range of infestation
1	*Barbastella barbastellus* (Schreber)	♀ 53	14 (26.4)	130	9.3 ± 8.2	2.5	1–42
		♂ 267	43 (16.1)	610	14.2 ± 10.2	2.3	1–115
		Σ 320	57 (17.8)	740	13 ± 9.9	2.3	1–115
2	*Eptesicus serotinus* (Schreber)	♀ 28	1 (3.8)	128	128 ± 23.8	4.6	128
		♂ 9	1 (11.1)	3	3 ± 0.5	0.3	3
		Σ 37	2 (5.4)	131	65.5 ± 20.8	3.5	3–131
3	*Myotis alcathoe* (von Helversen et Heller)	♀ 1	0	–	–	–	–
		♂ 8	1 (12.5)	1	1 ± 0.4	0.1	1
		Σ 9	1 (11.1)	1	1 ± 0.3	0.1	1
4	*M*. *bechsteinii* (Kuhl)	♀ 16	3 (18.8)	8	2.7 ± 6.5	0.5	1–4
		♂ 58	23 (39.7)	133	5.8 ± 5.1	2.3	1–30
		Σ 74	26 (35.1)	141	5.4 ± 5.0	1.9	1–30
5	*M*. *brandtii* (Eversmann)	♀ 29	2 (6.9)	2	1 ± 0.3	0.1	1
		♂ 85	3 (3.5)	92	30.7 ± 8.3	1.1	7–76
		Σ 114	5 (4.4)	94	18.8 ± 7.2	0.8	1–76
6	*M*. *daubentonii* (Kuhl)	♀ 51	7 (13.7)	15	2.3 ± 3.2	0.3	1–5
		♂ 216	25 (11.6)	89	3.6 ± 1.8	0.4	1–25
		Σ 267	32 (12)	104	3.3 ± 1.8	0.4	1–25
7	*M*. *myotis* (Borkhausen)	♀ 80	5 (6.3)	8	1.6 ± 0.5	0.1	1–4
		♂ 171	11 (6.4)	81	7.4 ± 3.1	0.5	1–31
		Σ 251	16 (6.4)	89	5.6 ± 2.6	0.4	1–31
8	*M*. *nattereri* (Kuhl)	♀ 214	11 (5.1)	61	5.5 ± 1.63	0.3	1–16
		♂ 473	89 (18.8)	653	7.3 ± 5.0	1.4	1–61
		Σ 687	100 (14.6)	714	7.1 ± 4.3	1	1–61
9	*Plecotus auritus* (L.)	♀ 34	8 (23.5)	62	8 ± 7.6	1.8	1–43
		♂ 140	47 (33.6)	565	12 ± 13.0	4	1–100
		Σ 174	55 (31.6)	627	11.4 ± 12.1	3.6	1–100
10	*P*. *austriacus* (Fischer)	♀ 0	–	–	–	–	–
		♂ 1	1 (100)	70	70	70	70
11	*Rhinolophus hipposideros* (Bechstein)	♀ 345	5 (8.3)	14	2.8 ± 0.5	0	2–7
		♂ 60	0	–	–	–	–
		Σ 405	5 (1.2)	14	2.8 ± 0.5	0.03	2–14
	Total	2339	300	2725			

### Seasonality in parasitism

**Fig. 2:**
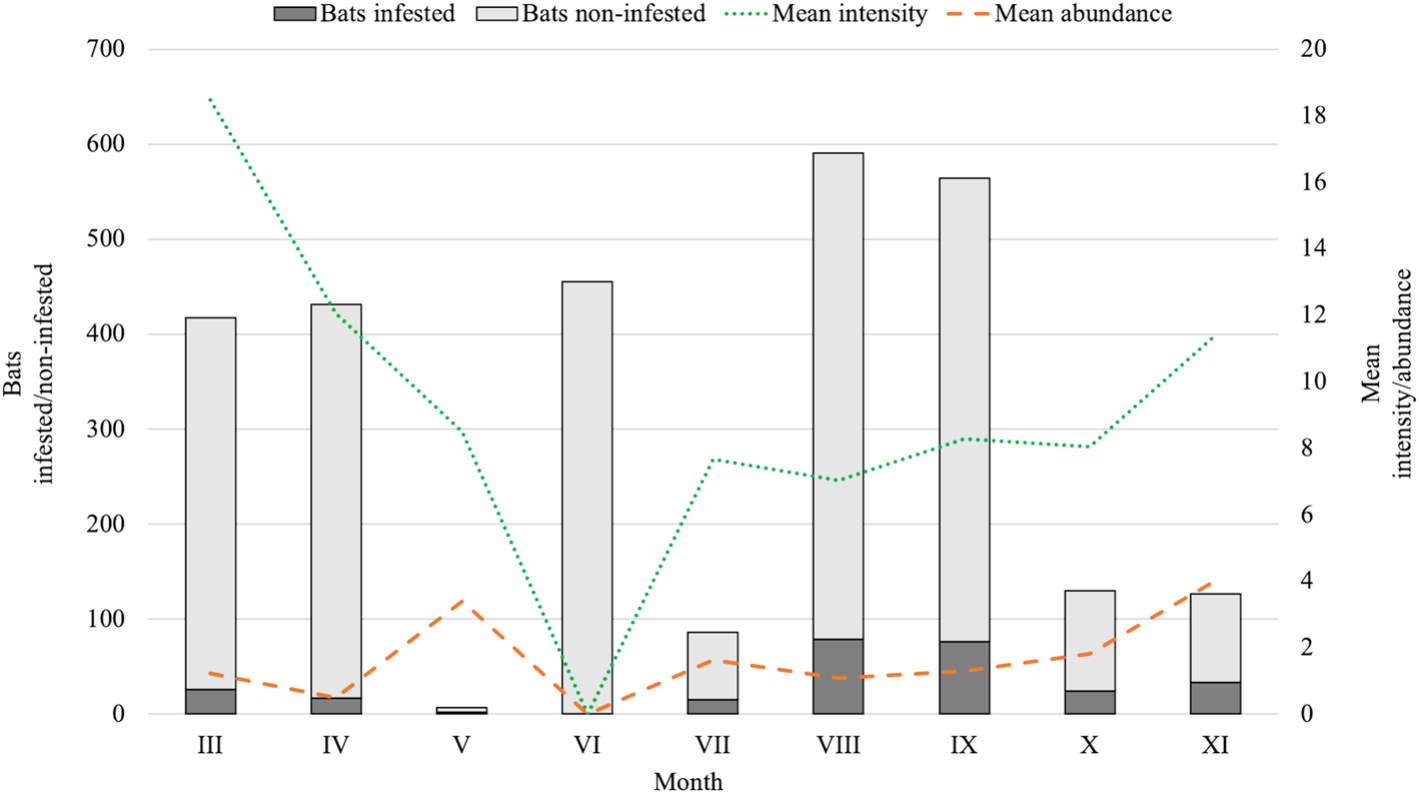
Trends in seasonal occurrence of chigger-infested bats in Poland. Data cumulated for the years 2015–2019, excluding the period of bat hibernation, between December and February

The only month in which no infested bats were observed was June (Fig. 2). The earliest appearance of unengorged trombiculids on Chiroptera was in July, with a tendency to shift the onset of appearance to late July/early August, depending on the host species. The highest total number of larvae collected was in autumn, which was related to the higher number of hosts caught due to bats swarming (Fig. 2). At that time the larvae were collected from 10 bat species, only not from *M*. *alcathoe*. In winter, with a limited collection of larvae from hosts during hibernation, bats with larvae were observed under non-used bridges, in tunnels, on rock shelfs, cave ceilings and walls, located close to the entrance of hibernacula. In March and April, at the increasing activity of bats, associated with their emergence from hibernation roosts, larvae were recorded on *B. barbastellus*, *M. brandtii*, *M. daubentonii*, *M. myotis*, *M. nattereri*, *P. auritus*, *P. austriacus*, and *R. hipposideros*. In bats caught in places more remote from hibernacula, a lower number of larvae was observed compared to those stated on hosts just emerging from roosts. Only one infested specimen of *M*. *alcathoe* was caught in mid-April in a park (recreational area). A remarkable decrease in the number of larvae was observed in the mid- and late spring (Fig. 2). In May larvae were observed on only two specimens of *M. nattereri*, in a mountainous locality in southern Poland, with lower temperatures recorded compared to other study areas. In the late spring and in summer, during the recurrent check of bat boxes and maternity roosts occupied by *M*. *nattereri*, *P. pipistrellus*, *P. nathusii*, *N. noctula* and *R*. *hipposideros*, the absence of larvae on these hosts was confirmed.

### Location of larvae on hosts’ body

The larvae were observed exclusively on the heads of hosts, including on the edges of earlobes, on tragi, and around the eyes, mouth, and nasal opening. The mites tended to form clusters on sparsely haired areas on the ears, whereas only single specimens were observed on the eyes, lips and nasal areas. The overall level of engorgement of chiggers was lower within clusters, in comparison with individuals that parasitized at a distance from each other. There was some variation among host species with regard to which parts of the ears had the most mite larvae. On *B. barbastellus* the larvae were aggregated at the edge of the earlobes and within the Henry’s pocket (cutaneous marginal pouch of the ear); on *M. bechsteinii* they were found on the inner and outer side of the earlobe; and on *P. auritus* and on the only specimen of *P. austriacus* caught during the survey they were at the edge of earlobes and on the tragi.

### Species identity of bat-associated chiggers

Based on morphological analyses, all larval trombiculids we examined belonged to the genus *Leptotrombidium*. Ten percent of specimens represented *L*. *russicum*. For other examined specimens the character states went beyond the variability known for *L. russicum*. Due to the mosaic distribution of diagnostic morphological traits, compared to the data on hitherto known members of the genus, the identification of these specimens to species level was not possible.

**Fig. 3:**
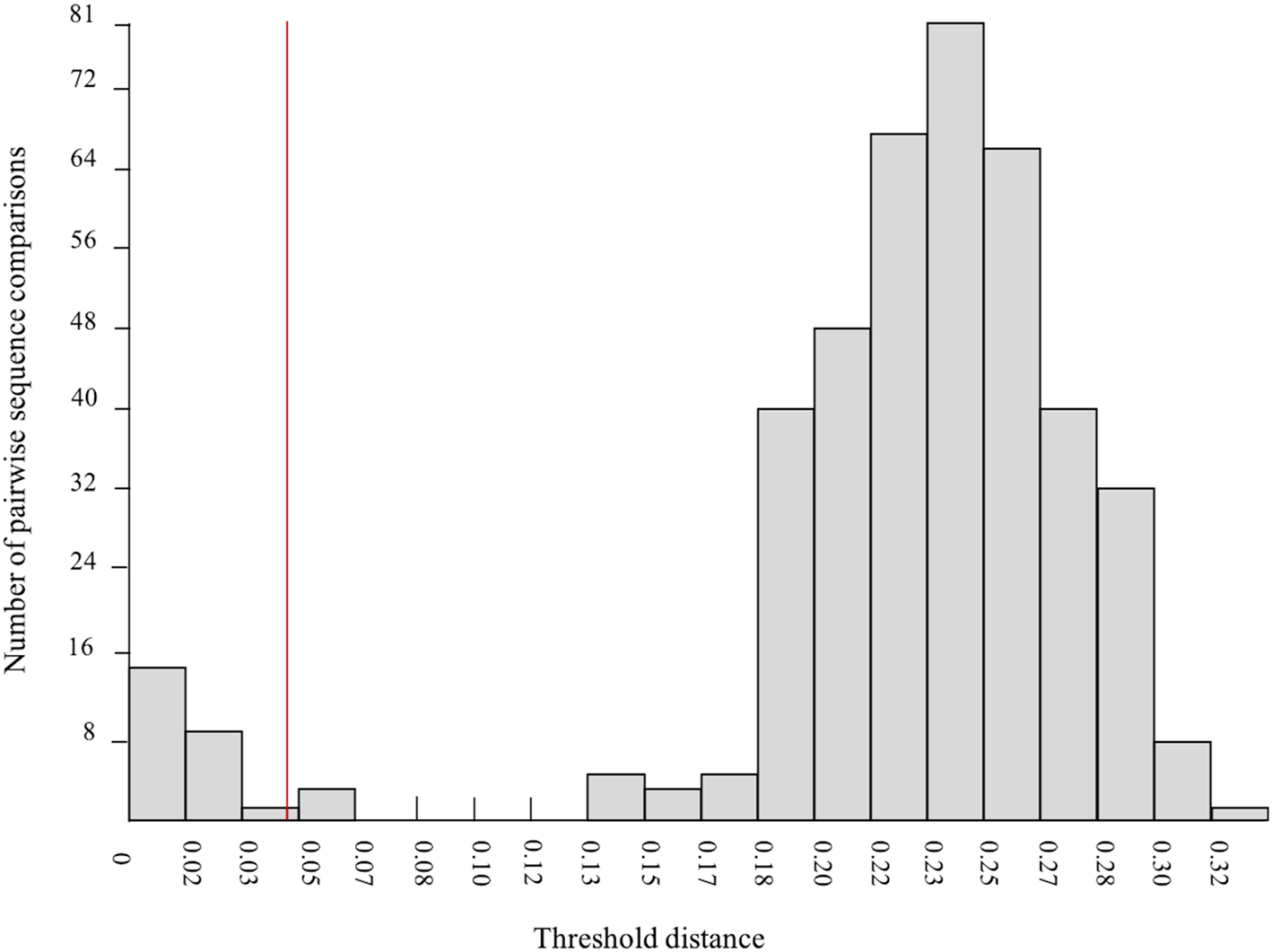
Distribution of K2P pairwise distances revealed by ASAP. Red line denotes threshold value of intraspecific divergence. (Color figure online)

**Fig. 4:**
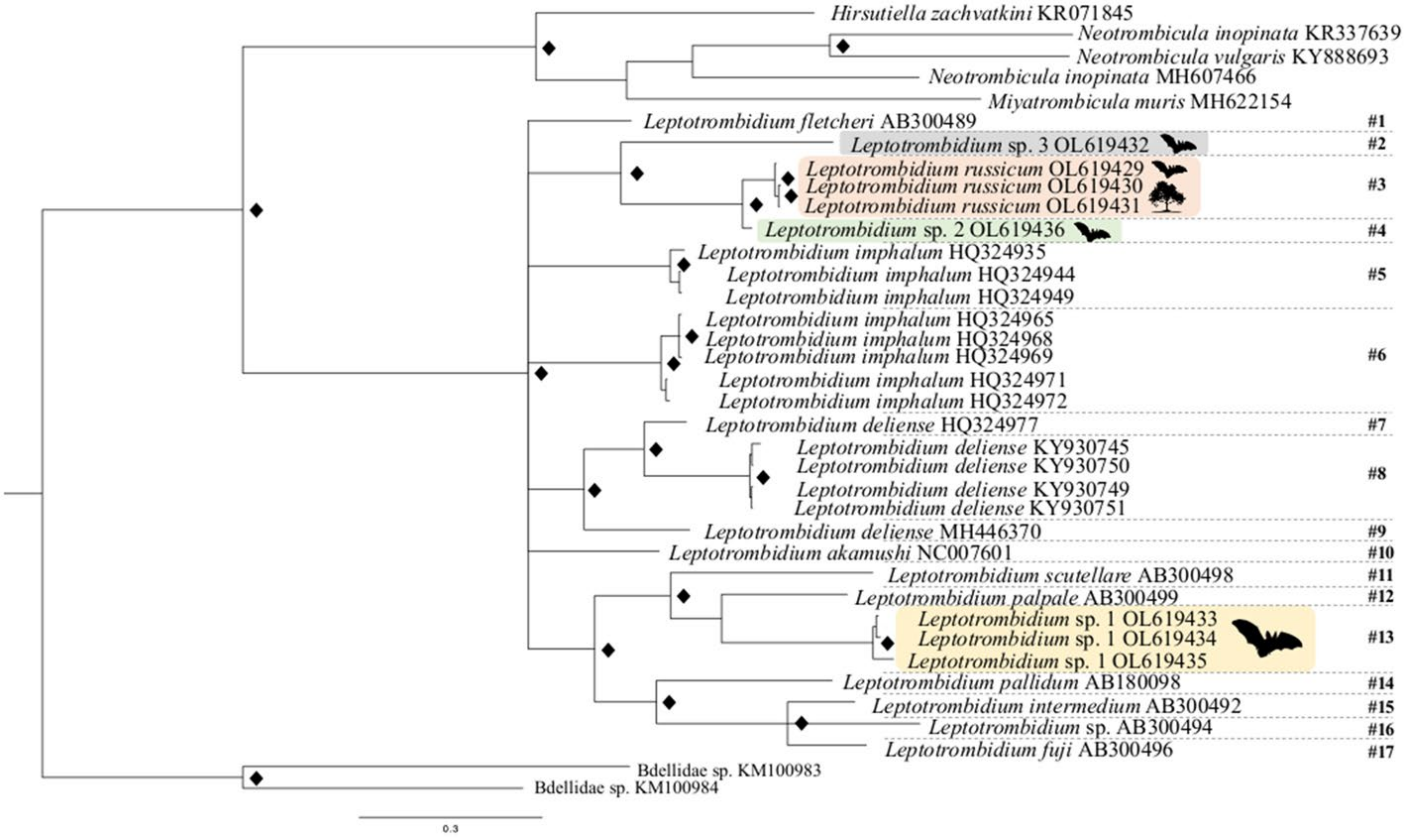
Phylogenetic tree (Bayesian inference, BI) based on COI dataset. Diamond symbols refer to Bayesian posterior probability (PP) of > 90%. Bat pictograms reflect cases in which larvae were collected from hosts, tree pictograms cases in which deutonymphs were collected from daily roost. Hash symbols (#) with numbers denote groups obtained using the ASAP method

The ASAP analyses of COI sequences from GenBank and eight specimens that we sequenced ourselves (n = 30 sequences in total, alignment length 540 bp) indicated the occurrence of 17 potential groups/species within *Leptotrombidium* (see # symbols in Fig. 4) (asap-score = 2; P = 0.20, W = 0.0112), with the threshold distance (d_T_) 4.6%. The barcode gap within *Leptotrombidium* revealed a p-distance between groups/species, ranging from 7 to 13% (Fig. 3). The sequences obtained from trombiculids collected in Poland were assigned to four independent groups. Group #3 contained three sequences (Fig. 4): OL619429 (obtained from larva collected from *B*. *barbastellus*), OL619430 and OL619431 (obtained from deutonymphs collected at the same daily roost). Group #13 also contained three sequences (Fig. 4): OL619433 (obtained under laboratory conditions from deutonymph which emerged from larva collected from *M*. *nattereri*), OL619434, OL619435 (obtained from larvae collected from *M*. *bechsteinii* and *M*. *daubentonii*, respectively). These groups correspond to the specimens assigned to *L. russicum* and to *Leptotrombidium* sp. 1, respectively, based on morphological criteria. Two other groups (Fig. 4; groups #4 and #2) contained one sequence each, OL619436 (from larva ex *M*. *nattereri*) and OL619432 (obtained, under laboratory conditions, from deutonymph which emerged from larva collected from *B*. *barbastellus*), identified as *Leptotrombidium* sp. 2 and *Leptotrombidium* sp. 3.

### Phylogenetic relationships

The BI phylogenetic tree (Fig. 4) corresponded with the results of ASAP. All branches received a high BI support. *L. russicum*, *Leptotrombidium* sp. 2 and *Leptotrombidium* sp. 3 formed a sub-clade separate from *Leptotrombidium* sp. 1. The genetic within-clade distance recorded for *L. russicum* was 0–1%. The genetic distance between the sequences of *L*. *russicum* and *Leptotrombidium* sp. 2 was 5.6–5.9%, between *L*. *russicum* and *Leptotrombidium* sp. 3 it was 27.6–28%, whereas the highest distance, 27.6–31%, was recorded between *L*. *russicum* and *Leptotrombidium* sp. 1, the latter revealing the sister relations with *L*. *palpale*.

## Discussion

Parasitism by trombiculid larvae was confirmed during our survey for the nine species of bats previously recorded as hosts of chiggers in Poland. In addition, the presence of chiggers on *Myotis myotis* constituted the first country record for this chigger-bat association, whereas a discovery of one larva on *M*. *alcathoe*, was the first observed case of this association in the world. Chiggers were not observed on eight of the bat species that were surveyed, of which *M. emarginatus* and *Pipistrellus nathusii* have never been recorded as hosts of Trombiculidae.

The different biology and ecology of the host species may translate into different abundance and different intensity and preferences of parasite towards the host’s sex (Marshall 1982; Freeland 1983; Hawlena et al. 2006; Krasnov et al. 2012). Chiroptera species associated with the forest ecosystem and most frequently infested by *Leptotrombidium* larvae were *B. barbastellus*, *M. nattereri* and *P. auritus*. These species start late summer/autumn activity relatively early (compared to other bat species) and have a varied number of activity peaks during swarming (Ignaczak et al. 2019) which indicates the potential opportunity created for larvae to infest the host at that time.

Our research revealed a male bias in bat-associated Trombiculidae. Previous reports of male-biased parasitism by bat-associated chiggers have been limited to few sources (Jones 1998; Poissant and Broders 2008). The results of studies on bat parasites (other than Trombiculidae) indicate a female-biased infestation (Marshall 1982; Schalk and Forbes 1997; Komeno and Linhares 1999; Chilton et al. 2000; Morales-Montor et al. 2004; Zahn and Rupp 2004; Lučan 2006; Christe et al. 2007; Frank et al. 2015; Postawa and Nagy 2016), whereas only a few studies indicate higher infestation in males (Moore and Wilson 2002; Morand et al. 2004; Sponchiado et al. 2015) or no correlation between the sex and the level of infestation (Moura et al. 2003; Czenze and Broders 2011). Frequent changes of roosts, solitary lifestyle, grooming, and the immune system seem to be of key importance in explaining the relatively low infestation of male bats with ectoparasites from various taxa (Kunz 1976; Moore 2013). On the other hand, the tendency to riskier behaviors in males (Schmid-Hempel 2011) may predict greater susceptibility to parasites (higher infestation), as stated for various mammalian hosts (Krasnov et al. 2012; Oliver-Guimerá et al. 2017).

The patterns of abundance and infestation are strictly associated with the complex life cycle and phenology of chiggers. The evolutionary relationships of trombiculid larvae with vertebrates, unlike most arthropod-associated Parasitengona, facilitated modifications in the phenology of species in a temperate zone, expressed in an extended time of larval appearance through prolonged contact with the host at unfavorable conditions. The highest number of larvae observed in this study at the turn of summer and autumn corresponds to the results of studies carried out on rodents in Poland (Moniuszko et al. 2015, 2017; Moniuszko and Mąkol 2016) and in Slovakia (Daniel 1961). The presence of larvae on bats in winter and in early spring, followed by the decrease in the number later in spring, indicates a gradual abandonment of the hosts at the time of transformation of chiggers from larvae to deutonymphs, until the complete absence of parasites, usually recorded in May.

Parasitic larvae were observed on bat species that most likely prefer places with dynamic microclimate during hibernation. While staying closer to the exit holes, the members of these species receive faster signals related to the increase in temperature outside the caves, which results also in an increase in body temperature and accelerates the termination of hibernation. The onset of bats’ activity is probably a factor that stimulates the activity of the larvae. The latter seems to be consistent with slight shift of this phenomenon in time, observed in larvae found on individuals hibernating in the deeper parts of the wintering places, with a more stable microclimate.

The data on chiggers’ tendencies to attach to particular parts of the bat's body are inconsistently reported and limited to side information in works devoted to other issues (Domrow 1962; Vercammen-Grandjean 1963; Brown 1997). We could observe that larvae generally attached to areas with limited hair cover, facilitating easy access to the skin. Specimens parasitizing individually or in small clusters were more engorged than those forming the larger clusters. The latter corroborates Goff's (1982) observations on rodent-associated chiggers. The parts of the bats' body occupied by the members of *Leptotrombidium* were analogous to those listed by Vercammen-Grandjean (1963), Harmata (1967), Haitlinger (1979), and Kalúz and Ševčík (2014). Variation among host species in the specific locations where the larvae attached to the host's ears may result from the different structures of the ears (shape, thickness, length, presence, or absence of a tragus) in different bat species. Traub and Wisseman (1974), Goff (1979), and Barnard et al. (2015) considered the host species as the main factor determining the anatomical site preferences of larvae. According to these authors, the grooming behavior, in the case of bats, consisting of licking and scratching (Zhang et al. 2013), may have a direct impact on the area where parasites are observed.

The gradual increase in species richness towards tropical latitudes is not directly reflected in the number of trombiculid species recorded from Central European countries, which is due to the uneven state of knowledge of the chigger fauna and the still unstable status of species separated based on the morphological characters. All specimens subjected to detailed morphological analyses in this study were assigned to *Leptotrombidium*. The genus comprises 342 named species worldwide, of which 23 are associated with Chiroptera (Stekolnikov 2013). Of those, only *L. russicum* has been previously recorded from Central Europe (Zajkowska et al. 2018). Our research indicated the presence of *L. russicum* on bats in Poland, and in addition, the presence of at least three other species within the same genus. The representatives of *Oudemansidium* were not confirmed in the examined material. This genus—with about 10 nominal species, two of which (*O. musca* and *O*. *komareki*) were reported from Central Europe—is considered as associated only with bats (Shatrov and Kudryashova 2008; Stekolnikov 2018). The absence of *O. musca*, previously collected from bats in Poland (Haitlinger and Ruprecht 1985; Haitlinger 1979; Haitlinger and Łupicki 2008) may indicate an accidental occurrence of this species in the country. Misidentification of *Oudemansidium* and *Leptotrombidium* can be excluded due to clear morphological differences between the members of these genera.

Our study revealed a lack of consistency between the morphological and molecular criteria for species discrimination in Trombiculidae. The current taxonomy of chiggers is based on morphology. Discrepancies in the selection of key characters and character states in identification keys constructed by different authors (Kudryashova 1998; Fernandes and Kulkarni 2003; Stekolnikov 2013) often lead to ambiguous decisions which may result in misidentification. With the priority given to quantitative traits—total number of dorsal and ventral setae (NDV), *index pedibus* (IP)—in species diagnoses, verification of the material based solely on these features is often insufficient to sanction species distinctness. The strategies of host selection, in which the habitat plays a crucial role in the formation of interactions (Peng et al. 2018; Lv et al. 2019), seem to differ from those observed in other representatives of terrestrial Parasitengona. Moreover, the host-associated differences in morphological traits (Moniuszko et al. 2015) at a relatively wide host spectrum, confirm the existence of various evolutionary modes among morphological traits in Trombiculidae. Thus, correct identification of trombiculid species, also influencing the inference about the host spectrum and host specificity, is not possible without referring to criteria beyond the currently employed morphological ones. Species delimitation through an integrative approach to taxonomy constitutes a solution in studies on Trombiculidae. Attention should be paid to the potential presence of cryptic species confirmed in other systematic groups of mites (de Rojas et al. 2002; Navia et al. 2013; Doña et al. 2015; Low et al. 2015). Use of non-morphological criteria in species discrimination of chiggers has been rare up to now. Korkusol et al. (2010) tried to develop a molecular taxonomic key for the precise identification of trombiculid mites using the COI gene; the results obtained for the deduced amino acid sequence of full-length COI revealed a surprising amount of variation for species identified earlier based on a morphological criterion. It is noteworthy, that the scale of genetic differences, also observed in this study, is not universal. Intraspecific variability for selected species of insects, spiders, and mites, as a rule, does not exceed 3.6%, whereas interspecific variability is in the range of 2.3–29.9% (Anderson and Morgan 2007; Dabert et al. 2008; Skoracka and Dabert 2010; Iftikhar et al. 2016; Mąkol et al. 2019). In the present study, the intraspecific variation threshold in Trombiculidae attained 4.6%. The value of interspecific distance (> 20%) may, however, pose a question on the common generic identity of putative species examined in the present survey.

Our research reveals several still open issues and problems related to the taxonomy, ecology, and biology of Trombiculidae, with special reference to interaction of chiggers with their host, and implies the need for deeper insight into this family, with the application of hitherto neglected tools.

## Data Availability

Data supporting the conclusions of this article are included in the article.

## Code availability

Sequences used in this study are deposited in the GenBank database under the accession numbers: OL619429, OL619430, OL619431, OL619432, OL619433, OL619434, OL619435 and OL619436.
